# IL-32 and IL-17 interact and have the potential to aggravate osteoclastogenesis in rheumatoid arthritis

**DOI:** 10.1186/ar4089

**Published:** 2012-11-13

**Authors:** Young-Mee Moon, Bo-Young Yoon, Yang-Mi Her, Hye-Joa Oh, Jae-Seon Lee, Kyoung-Woon Kim, Seon-Yeong Lee, Yun-Ju Woo, Kyung-Su Park, Sung-Hwan Park, Ho-Youn Kim, Mi-La Cho

**Affiliations:** 1 The Rheumatism Research Center, Catholic Research Institute of Medical Science, The Catholic University of Korea, 505 Banpo-dong, Seocho-gu, Seoul 137-701, South Korea; 2 Department of Internal Medicine Inje University Ilsan Paik Hospital, Juhwa-ro 170, Ilsanseo-gu, Goyang-si, Gyeonggi-do 411-706, South Korea; 3 Conversant Research Consortium in Immunologic disease, Seoul St. Mary's Hospital, College of Medicine, The Catholic University of Korea, 505 Banpo-dong, Seocho-gu, Seoul 137-701, South Korea

## Abstract

**Introduction:**

Interleukin (IL)-32 and IL-17 play critical roles in pro-inflammatory responses and are highly expressed in the synovium of patients with rheumatoid arthritis (RA). We investigated the relations between these two cytokines (IL-17 and IL-32) for their ability to induce each other and to stimulate osteoclasts in RA fibroblast-like synoviocytes (FLSs) and T cells.

**Methods:**

FLSs were isolated through surgical synovectomy obtained from patients with RA or osteoarthritis (OA). Real-time PCR were performed to evaluate the expression of IL-32, IL-17 and osteoclast-related genes. Immunohistochemical staining and tartrate-resistant acid phosphatase (TRAP) staining were performed to determine the distribution of inflammatory cytokines and the presence of osteoclastogenesis.

**Results:**

IL-17 induced the expression of IL-32 in the FLSs from RA patients, as assessed by microarray. IL-32 production was increased by IL-17. IL-32 in the FLSs from RA patients induced the production of IL-17 in CD4^+ ^T cells. IL-32 and IL-17 were colocalized near TRAP-positive areas in joint specimens. IL-17 and IL-32 synergistically induced the differentiation of osteoclasts, as demonstrated by the expression of osteoclast-related genes. IL-32 and IL-17 also could induce resorption by osteoclasts in a RANKL-dependent manner.

**Conclusions:**

IL-17 affected the expression of IL-32 in FLSs of RA patients and IL-32 induced the production of IL-17 in CD4+ T cells. Both IL-17 and IL-32 cytokines can reciprocally influence each other's production and amplify the function of osteoclastogenesis in the in RA synovium. Separately, IL-17 and IL-32 each stimulated osteoclastogenesis without RANKL. Together, the two cytokines synergistically amplified the differentiation of osteoclasts, independent of RANKL stimulation.

## Introduction

Rheumatoid arthritis (RA) is a chronic systemic autoimmune disease that predominantly affects multiple peripheral synovial joints. The synovial environment has numerous inflammatory cells such as T cells, B cells, fibroblast-like synoviocytes (FLSs) and antigen-presenting cells, which can cause the development of RA. FLSs constitute the synovial lining cells that have a key role in pannus formation and destruction of joints [1]. In addition, numerous cytokines have been implicated in the immune processes that are associated with RA. T cells, the most invading type of lymphocyte in the RA synovium, can contact and activate FLSs [2,3]. A variety of *in vitro *and *in vivo *models have shown that TNF-dependent networks are involved in critical pathogenic interactions in RA synovitis. In recent years, new novel cytokines such as IL-17 and IL-32 have been reported to be involved in the pathogenesis or regulation of synovial inflammation.

IL-17, a proinflammatory cytokine produced by T helper (Th)17 cells [4], plays a key role in the propagation of joint inflammation, cartilage destruction and bone erosion[5,6], and it is present in both the synovium and synovial fluid [7]. IL-17 participates in the joint inflammation of RA via activation of T cells and FLSs by secreting cytokines and chemokines such as IL-6, IL-8, 1L-16, stromal cell-derived factor-1 (SDF-1), matrix metalloproteinase (MMP)-3 and MMP-1 [8-11]. Moreover, IL-17 is well known as a strong inducer of osteoclastogenesis [5].

IL-32, a recently discovered cytokine, was originally described as natural killer (NK) cell transcript 4 (NK4) [12]. IL-32 has four splice variants, *IL-32α, IL-32β, IL-32δ *and *IL-32γ*, with *IL-32α *as the most abundant transcript, and *IL-32γ *as the most active isoform. The expression of TNF-α and IL-6 are significantly correlated with IL-32γ expression [13]. IL-32 is expressed in NK cells, T cells, epithelial cells and blood monocytes upon stimulation by inflammatory cytokines such as IL-1β, IL-18, IFN-γ and TNF-α by the phosphatidylinositol 3-kinase/Akt and NF-κB/AP-1 systems [14]. IL-32 is highly expressed in the synovial tissue and FLSs of RA patients, but not in osteoarthritis patients [15]. IL-32 can also induce inflammatory cytokines and chemokines such as TNF-α, IL-1β, IL-8 and IL-6 by the activation of NF-κB and p38 mitogen-activated protein kinase [16]. Injection of human IL-32 into the knee joints of naïve mice results in joint swelling, infiltration and cartilage damage. For these reasons, IL-32 has been recognized as a proinflammatory cytokine, and has been implicated in inflammatory disorders such as RA and inflammatory bowel disease [17,18].

IL-32 and IL-17 are thought to be associated with pathogenesis, and are frequently mentioned together as they seem to have similar roles. It was reported that CXC chemokine receptor 4 (CXCR4), lamina propria lymphocytes (LPL) and IL-32 were identified by IL-17A or IL-17F plus TNFα on RA synoviocytes [19]. Studies using RA FLSs and CD4^+ ^T cells or dendritic cells have shown a reciprocal induction between TNFα and IL-32, creating a TNFα/IL-32/TNFα-positive autoinflammatory loop [20]. Moreover, IL-32 production is partially dependent on TNFα, and the treatment of RA patients with anti-TNFα has resulted in the reduction of IL-32 protein in synovial tissue. In a recent report, it was suggested that IL-32γ contributes to the maturation and activation of immature dendritic cells (DCs) and increases Th1 and Th17 response by IL-12 and IL-6 [21]. In addition, IL-17 and IL-32 have been shown to influence pathogenesis via the common protein p300 and DAPK-1, through the TNF-R1 dependent/independent pathway [22].

From these investigations, we hypothesized that IL-32 and IL-17 interact with each other, and function to amplify inflammatory reactions in RA. In this study, we examined the interaction between the two cytokines, and further investigated their synergistic involvement in osteoclastogenesis functions. Osteoclasts have a key role in the joint destruction of RA. It was reported that both IL-17 and IL-32 induce the generation of osteoclasts [5,23,24]. IL-17 functionally upregulates the receptor activator of NF-κB (RANK) on osteoclast precursors causing increased sensitivity to RANK ligand (RANKL) signaling, leading to increased bone destruction [25]. A recent study showed that IL-32γ has a greater potential for generating osteoclasts compared to IL-17 in the presence of soluble RANKL [24].

Our results suggest that IL-17 and IL-32 stimulate each other's production, and both inflammatory cytokines synergistically induce osteoclastogenesis. Eventually, IL-17 and IL-32 might accelerate synovial inflammation and erode cartilage and bone by osteoclastogenesis in patients with RA.

## Materials and methods

### Patients and mice

FLS cell lines were prepared from the synovectomized tissue of RA patients who were undergoing joint replacement surgery [8]. Six- to eight-week-old male DBA/1J mice () were maintained for CIA induction. *IL-1R *antagonist-deficient mice (*IL-1Ra-/- *mice) in a BALB/c background were provided by Dr Y Iwakura (University of Tokyo, Tokyo, Japan). All the experimental procedures were examined and approved by the Animal Research Ethics Committee at the Catholic University of Korea.

### Cell preparation

Peripheral blood was obtained from healthy donors using heparin-treated syringes. Peripheral blood mononuclear cells (PBMCs) were isolated by density centrifugation using Ficoll-Hypaque (Pharmacia LKB, Uppsala, Sweden). Mice splenocytes were isolated through a mesh and the red blood cells (RBCs) were lysed with 0.83% ammonium chloride. To purify the CD4^+ ^T cells, the cell suspensions were incubated with CD4-coated magnetic beads (Miltenyi Biotec, Bergisch Gladbach, Germany) for 15 minutes at 4°C and the cells were isolated on magnetic-activated cell sorting (MACS) separation columns (Miltenyi Biotec). The CD4^+ ^T cells were cultured with the stimuli: recombinant human IL-17, human IL-23, human IL-32α (R&D systems, Minneapolis, MN, USA), TGF-β (Peprotech, Rocky Hill, NJ, USA), and membrane-bound anti-CD3 antibody (0.5 μg; BD PharMingen, CA, USA), and the cells were pretreated with the inhibitors parthenolide (10 μM), LY294002 (10 μM) (A.G. Scientific, Inc., San Diego, CA, USA), or an anti-human IL-17 blocking antibody (R&D systems) for 2 h.

### Preparation of an autoimmune arthritis mice model

To induce type ll collagen-induced arthritis (CIA), 0.1 ml of an emulsion containing 100 μg bovine type II collagen (CII) and complete Freund's adjuvant (CFA; Chondrex, Redmond, WA, USA) was injected intradermally into the base of the tail as a primary immunization. Two weeks later, a booster injection of 100 ug CII dissolved and emulsified 1:1 with incomplete Freund's adjuvant (DIFCO, Detroit, MI, USA) was administered to the hind leg.

### RNA preparation and real-time PCR

The total RNA was extracted using TRI Reagent (MRC, Cincinnati, OH, USA) according to the manufacturer's instructions. The RNA concentrations were measured using a NanoDrop ND-1000 (Thermo Fisher Scientific, Waltham, MA, USA). Reverse transcription of 2 μg of the total mRNA was conducted at 42°C using RevertAid™ M-MuLV Reverse Transcriptase and RNase inhibitor (Fermantas, Burlington, ON, Canada). PCR amplification of cDNA aliquots was performed by adding SYBR green mixture (Takara, Shiga, Japan) in a LightCycler (Roche Diagnostics Mannheim, Germany). The relative expression levels were calculated by normalizing the targets to the endogenously expressed housekeeping gene (β-actin). Melting curve analysis was performed immediately after the amplification protocol under the following conditions: 0 s (hold time) at 95°C, 15 s at 65°C and 0 s (hold time) at 95°C. The temperature change rate was 20°C/s except in the final step, in which it was 0.1°C/s. The crossing point (Cp) was defined as the maximum of the second derivative from the fluorescence curve.

### Bead array gene expression analysis

Total RNA (200 ng of total RNA) was used as a template for producing double-stranded cDNA and to perform *in vitro *transcription amplification using the Illumina Total Prep RNA amplification kit (Ambion, TX, USA), following the manufacturer's instructions. The biotin-labeled cRNA (750 ng) was purified and hybridized to the HumanRef-8 BeadChip at 58°C for 16 h by following the Illumina whole-genome gene expression protocol for BeadStation. The arrays were scanned with the Illumina BeadArray Reader. Data normalization was performed using quantile normalization.

### Histology and Immunohistochemistry

Immunohistochemical staining was performed on sections of the synovium. Briefly, the synovial samples, obtained from four patients with RA and one patient with OA, were fixed in 4% paraformaldehyde solution overnight at 4°C, dehydrated with alcohol, washed, embedded in paraffin and sectioned into 7-μm-thick slices. The sections were depleted of endogenous peroxidase activity by adding methanolic hydrogen peroxide (H_2_O_2_) and blocking with normal serum for 30 minutes. The sections were then incubated overnight at 4°C with goat anti human IL-17, anti -IL-32 antibody (R&D Systems), anti -human NF-κB p50 and 65 (Santa Cruz Biotechnology, Santa Cruz, CA, USA) and p-IκB, p-AKT and AKT (Cell Signaling Technology, Danvers, MA, USA). A hind leg of each mouse was fixed with 1% formalin, decalcified in EDTA and embedded in paraffin wax. The sections were then stained with H&E and tartrate-resistant acid phosphatase (TRAP) stain. The tissues were incubated with the primary IL-17 antibody (Santa Cruz Biotechnology) and IL-32 antibody (Abfrontier, Seoul, South Korea) overnight at 4°C. The samples were incubated with biotinylated anti-goat IgG and anti-rabbit IgG secondary antibodies for 20 minutes. The sections were then incubated with streptavidin-peroxidase complex (Vector Laboratories Ltd., Peterborough, UK) for 1 h followed by incubation with 3, 3-diaminobenzidine (Dako, Glostrup, Denmark). The sections were counterstained with hematoxylin and the samples were photographed using a photomicroscope (Olympus, Tokyo, Japan).

### Osteoclast differentiation and activity

After isolation from whole blood, human PBMCs were incubated for 3 h. To remove nonadherent cells, the cultures were rinsed in medium. The adherent cells were cultured as osteoclast precursors using recombinant human macrophage-colony stimulating factor (M-CSF) in minimum essential medium alpha modification (α-MEM; Invitrogen, Carlsbad, CA, USA) and 10% heat-inactivated fetal bovine serum (FBS) for the first 3 days. The osteoclast precursors were rinsed in medium and stimulated with M-CSF and RANKL, or IL-17 or IL-32. All the factors were replenished every 3 days and the cultures were maintained for up to 21 days. A commercial TRAP kit (Sigma-Aldrich, St Louis, MO, USA) was used according to the manufacturer's instructions, and cells were counterstained with hematoxylin. TRAP-positive cells containing three or more nuclei were scored as osteoclasts. The TRAP-positive multinucleated cells (MNCs) were counted three times without the examiner having knowledge of the previously counted numbers of osteoclasts. To assess osteoclast activity, cell culture was performed as described above with dentine discs (IDS Inc., Boldon, UK) in 96-well plates. The cells were cultured for 21 days. At day 21 cells were removed from dentine discs using 10% sodium hypochlorite solution rinsed in distilled water. The dentin discs were then stained with 50% hematoxylin rinsed in distilled water. Resorption area was evaluated by light microscopy and measured using the TMOMRO analysis Ts Lite Image program (Olympus, Munster, Germany).

### Enzyme-linked immunosorbent assay (ELISA)

Antibodies for mouse IL-17 and human IL-17 were obtained from R&D Systems. The IL-17 concentration in the culture supernatants was measured by sandwich ELISA, according to the manufacturer's instructions. A standard curve was drawn by plotting the optical density versus the log of the concentration of IL-17.

### Flow cytometry analysis

Cells were stimulated for 4 h with phorbol 12-myristate 13-acetate and ionomycin. The cells were permeabilized using a cytoperm/cytofix kit (BD PharMingen) For surface staining, 5 × 10^5 ^cells were washed twice with PBS and stained with peridinin chlorophyll protein-cyanine 7.7-conjugated anti-human or mouse CD4 (BD PharMingen) for 30 minutes at 4°C. To measure the intracellular IL-17 concentrations, the cells were fixed and stained with fluorescein isothiocyanate-conjugated anti-human or anti-mouse IL-17 monoclonal antibody (BD PharMingen) for 30 minutes at 4°C. Staining for the isotype controls was performed simultaneously using an isotype control antibody (BD PharMingen). The cells were analyzed on a fluorescence-activated cell sorter (FACS), Calibur (BD). The events were collected and analyzed with FlowJo software (TreeStar, Ashland, OR, USA).

### Statistical analysis

The experimental values are presented as means ± SD. Statistical significance was determined by analysis of variance (ANOVA) with Bonferroni's post-test correction or Student's *t*-test, using the SPSS program (version 10.0); *P*-values < 0.05 were considered statistically significant.

## Results

### Microarray analysis of IL17A inducible cytokine- and chemokine-related genes

We utilized a microarray to compare the multiple gene expression profiles representative of the FLSs from patients with RA and the IL-17-stimulated FLSs. Table 1 shows the IL-17-inducible cytokine and chemokine genes in the FLSs from patients with RA. Several inflammation-related genes were highly expressed in the IL-17-stimulated FLSs from RA patients. Our microarray results indicated that IL-17A induced IL-32 expression (4.3-fold) in FLSs of RA patients (Table 1). Therefore, we examined whether IL-17 and IL-32 have an effect on each other in the inflammatory environment.

**Table 1:** Interleukin (IL)-17A-inducible genes as determined by microarray analysis of fibroblast-like synoviocytes (FLSs) from patients with rheumatoid arthritis (RA)

Accession numbers	Full name	Symbol	Folds increased
NM_000584.2	interleukin 8	*IL8*	509.5
NM_004591.1	chemokine (C-C motif) ligand 20	*CCL20*	382.0
NM_002993.2	chemokine (C-X-C motif) ligand 6	*CXCL6*	314.1
NM_000600.1	interleukin 6	*IL6*	120.6
NM_001511.1	chemokine (C-X-C motif) ligand 1	*CXCL1*	88.6
NM_007115.2	tumor necrosis factor, alpha-induced protein 6	*TNFAIP6*	21.1
NM_002089.1	chemokine (C-X-C motif) ligand 2	*CXCL2*	20.4
NM_002982.3	chemokine (C-C motif) ligand 2	*CCL2*	20.0
NM_005623.2	chemokine (C-C motif) ligand 8	*CCL8*	17.5
NM_002421.2	matrix metallopeptidase 1	*MMP1*	16.1
NM_001078.2	vascular cell adhesion molecule 1	*VCAM1*	15.2
NM_000201.1	intercellular adhesion molecule 1	*ICAM1*	9.3
NM_001565.1	chemokine (C-X-C motif) ligand 10	*CXCL10*	8.2
NM_001012632.1	interleukin 32	*IL32*	4.3
NM_002006.3	fibroblast growth factor 2	*FGF2*	2.7

Bead array gene expression analysis of FLSs and IL-17 activated FLSs from patients with RA (RA FLS) at 12 h. After serum starvation for 24 h, the RA FLSs were stimulated with IL-17A (20 ng/ml) or without IL-17A. Total RNA from RA FLSs was used for the Bead array gene expression analysis. The genes differentially expressed between the IL-17 activated RA FLSs and the RA FLSs were identified by ANOVA with a *P-*value < 0.01.

### IL-17 induced IL-32 expression via NF-κB and PI3kinase in the FLSs of patients with RA

**Figure 1:**
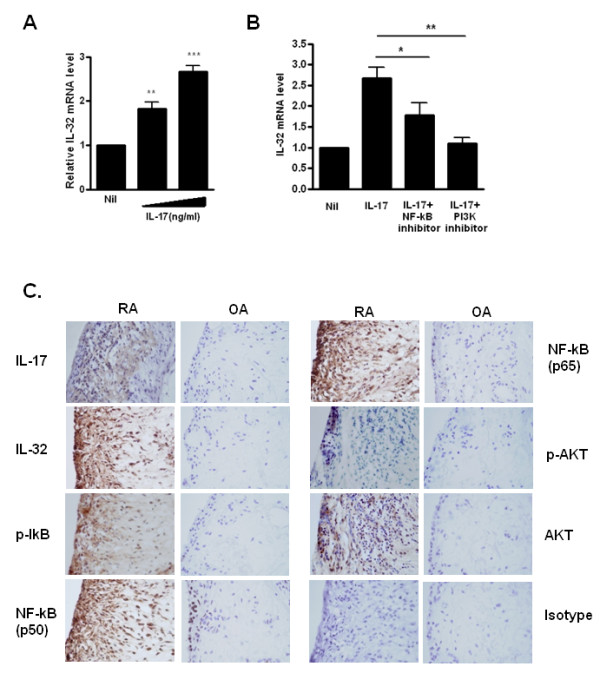
**IL-17 induced expression of interleukin (IL)-32 via NF-κB and PI3 kinase in fibroblast-like synoviocytes (FLSs) from patients with rheumatoid arthritis (RA)**. (**A**) FLSs from RA patients (RA FLSs) were cultured with increasing doses (1 and 5 ng/ml) of IL-17 for 12 h. IL-32 mRNA levels were measured by real-time PCR. ***P < 0.001 (in comparison with nil), ** *P *< 0.01 (in comparison with nil). (**B**) RA FLSs were pretreated with the signal inhibitors parthenolide (10 μM) or LY294002 (10 μM) for 2 h and then cultured with IL-17 (10 ng/ml) for 12 h. The IL-32 mRNA level was measured by real-time PCR. *P < 0.05 (in comparison with IL-17), **P < 0.01 (in comparison with IL-17). A and B show the means ± SD of more than three separate experiments. (**C**) Expression of IL-17, IL-32, phospho-IkB (p-IkB), NF-κB (p50), NF-κB (p65), phospho-Akt (p-Akt) and AKT in the synovium of patients with RA or osteoarthritis (OA). Immunostaining was performed using specific antibodies. Data are representative of three experiments with similar results.

FLSs obtained from synovial tissue of patients with RA during surgical synovectomy were stimulated by IL-17, and the IL-32 mRNA level was measured. The IL-32 mRNA level was increased in a dose-dependent manner (Figure 1A). The IL-32 mRNA level was decreased by the PI3K inhibitor LY294002 and the NF-κB inhibitor parthenolide, and PI3K and NF-κB molecules were associated with the IL-17-induced IL-32 production (Figure 1B) [13]. A higher level of IL-32 was expressed by IL-17-stimulated FLSs from patients with RA than from patients with OA. Similarly, NF-κB and PI3K signal molecules were also more highly expressed in synovium from patients with RA than from patients with OA (Figure 1C).

**Figure 2:**
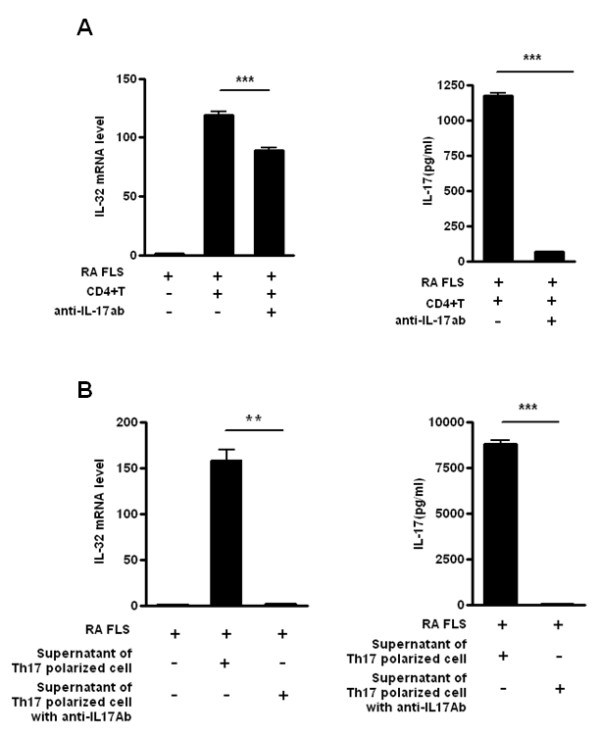
**Interleukin (IL)-17/T helper (Th)17-induced IL-32 expression from rheumatoid arthritis (RA) patients**. (**A**) Production of IL-32 by RA fibroblast-like synoviocytes (FLSs) in contact with CD4^+ ^T cells. RA FLSs and CD4^+ ^T cells from healthy donors were co-cultured. FLSs (1 × 10^5^) were cultured with CD4^+ ^T cells (1 × 10^6^) with or without anti-IL-17 blocking antibody (10 μg/ml). IL-17 production was then measured by sandwich ELISA, and the IL-32 mRNA levels of RA FLSs were determined by real-time PCR. ***P < 0.001 (compared with FLS+CD4^+ ^T cells). (**B**) Induction of RA FLS IL-32 production by the supernatant of Th17-polarized cell cultures. RA FLSs and the culture supernatants of Th17-polarized cells from healthy donors were co-cultured. CD4^+ ^T cells were incubated with membrane-bound anti-CD3 antibody (2 μg/ml), IL-6 (5 ng/ml), IL1β (5 ng/ml), IL-23 (10 ng/ml), TGF-β (5 ng/ml) with or without an anti-IL-17 blocking antibody incubated for 2 h before the next incubation) for 3 days to induce Th17.polarization. FLS (1 × 10^5^) were cultured with the culture supernatants of these Th17 polarized cells. IL-17 production was measured by sandwich ELISA and the IL-32 mRNA levels in RA FLSs were determined by real-time PCR. **P < 0.01 (compared with FLSs + culture supernatant of Th17 cells), *** *P *< 0.001 (compared with FLSs + culture supernatant of Th17 cells). The data are representative of three experiments with similar results.

In the inflammatory condition of RA synovitis, contact between T cells and FLSs is an important and necessary mechanism [26]. Co-incubation of FLSs from RA patients with CD4^+ ^T cells caused an increase in IL-32 mRNA levels in FLSs (Figure 2A, left panel) and an increase in IL-17 levels in the supernatants of co-cultures (Figure 2A, right panel). When treated with IL-17 blockade antibody under the same conditions, the IL-32 mRNA levels in the FLSs were suppressed (Figure 2A, left panel). The IL-17 levels in the supernatant of co-cultures were also markedly decreased by blocking with anti-IL-17 antibody (Figure 2A, right panel).

To confirm the role of IL-17 of CD4^+ ^T cells in induction of IL-32 expression in FLSs, the IL-17-rich supernatant from Th17-polarized cells was added to FLSs from RA patients. The IL-32 mRNA level in the FLSs from RA patients was increased in the presence of the Th17-polarized cell supernatant, and blocked with IL-17 blockade antibody (Figure 2B, left panel). Therefore, IL-17 derived from direct contact between FLSs from RA patients and CD4^+ ^T cells (Figure 2A), as well as that secreted by Th17-polarized cells (Figure 2B), can induce IL-32 expression in FLSs from RA patients. In both cases, IL-32 expression was arrested by IL-17 blockade. These results demonstrate that IL-17, an important cytokine in RA, has a direct role in IL-32 expression by the FLSs of RA patients.

### IL-32 induced the production of IL-17 in human CD4^+ ^T cells and differentiation of Th17 cells

**Figure 3:**
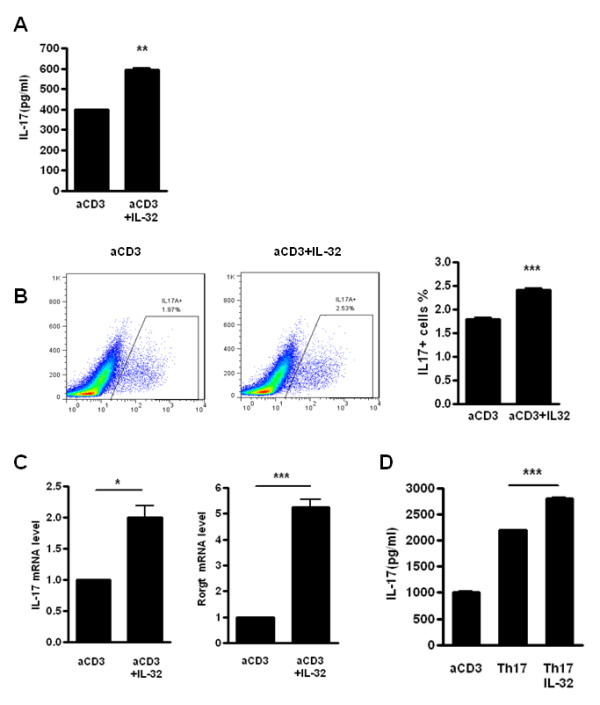
**Interleukin (IL)-32 induces the production of IL-17 in human CD4^+ ^T cells**. (**A**) CD4^+ ^T cells from healthy donor human peripheral blood mononuclear cells (PBMCs) were cultured with membrane-bound anti-CD3 antibody and with/without recombinant human IL-32 (5 ng/ml) for 3 days. IL-17 production was measured by sandwich ELISA. **P < 0.01 (compared with anti-CD3). (**B**) Flow cytometry analysis of the expression of IL-17A by CD4^+ ^T cells treated with membrane-bound anti-CD3 antibody (0.5 μg/ml) and with/without IL-32 (5 ng/ml) for 3 days. These cells were stained with anti-CD4-Percp cy7.7 and anti-IL-17-FITC, to determine the percentage of IL-17^+ ^cells in the CD4^+ ^gated population. *** *P *< 0.001 (compared with anti-CD3). (**C**) IL-17 and RORγt mRNA levels were measured by real-time PCR in stimulated CD4^+ ^T cells with a membrane-bound anti-CD3 antibody with/without IL-32 (5 ng/ml). * *P *< 0.05 (compared with anti-CD3), *** *P *< 0.001 (compared with anti-CD3). (**D**) CD4^+ ^T cells from healthy donors were cultured with membrane-bound anti-CD3 antibody (0.5 μg/ml), anti-CD28 (1 μg/ml), anti-IL-4 (2 μg/ml), anti-IFN-γ (2 μg/ml), IL-1β (20 μg/ml) and IL-6 (20 ng/ml) to induce Th17 polarization, and with/without recombinant human IL-32 (5 ng/ml) for 3 days. *** *P *< 0.001 (compared with Th17) (**A**), (B, left panel) and (**D**) are representative of three experiments with similar results. (**B**, right panel) and (**C**) represent means ± SD of three separate experiments.

Because IL-32 can induce inflammatory cytokines, we next assessed the IL-17 production by IL-32. To determine whether IL-32 induces IL-17 production, CD4^+ ^T cells from human PBMCs were cultured with membrane-bound anti-CD3 antibody to activate TCRs and the expression of IL-17 increased when was stimulated with IL-32 (Figure 3A). CD4^+ ^T cells from healthy donors were differentiated using anti-CD3, anti-CD28, anti-IL-4, and anti-IFN-γ, antibodies, and IL-1β and IL-6. FACS analysis showed an increase in IL-17-expressing cells after IL-32 stimulation (Figure 3B). In addition, IL-17 mRNA levels in human CD4^+ ^T cells were increased by IL-32 stimulation, and expression of RORγt, a transcription factor for Th17 differentiation, was also increased by IL-32 stimulation (Figure 3C). Th17 cells might secrete increased IL-17 in the presence of IL-32. Indeed, recombinant human IL-32 promoted IL-17 production (Figure 3D). These results suggest that IL-32 production is affected by IL-17 stimulation, and that IL-32 plays a role in both Th17 cell differentiation and IL-17 secretion.

### IL-32 induced IL-17 production in autoimmune arthritis mouse models

**Figure 4:**
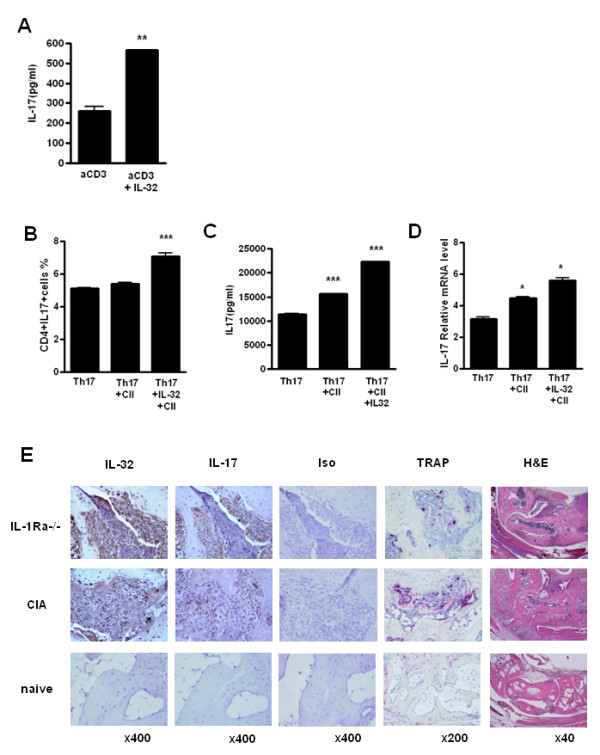
**Interleukin (IL)-32 induces IL-17 production in an autoimmune arthritis mouse model**. (**A**) CD4^+ ^T cells were isolated from the spleens of collagen-induced arthritis (CIA) mice, an autoimmune arthritis model. The cells were cultured with membrane-bound anti-CD3 (0.5 μg/ml) and with/without IL-32 (5 ng/ml) for 3 days. IL-17 production was measured by sandwich ELISA. ** *P *< 0.01 (compared with anti-CD3). (**B**-**D**) Expression of IL-17A by CD4^+ ^T cells cultured under Th17-polarizing conditions for 3 days: anti-CD3 (0.5 μg/ml), anti-CD28 (1 μg/ml), anti-IFN-gamma (2 μg/ml), anti-IL-4 (2 μg/ml), anti-IL-2 (2 μg/ml), IL-6 (20 ng/ml), TGF-beta1 (2 ng/ml) with/without IL-32 (5 ng/ml) and/or type ll collagen with irradiated antigen-presenting cells, and then stimulated for 4 h with PMA and ionomycin, followed by intracellular cytokine staining. The percentage of IL-17^+ ^cells in the CD4^+ ^gated population (**B**), *** *P *< 0.001 (compared with Th17) and cytokine level (**C**) were measured by sandwich ELISA, *** *P *< 0.001 (compared with Th17) and mRNA levels (**D**) were measured by real-time PCR, * *P *< 0.05 (compared with Th17). **B**-**D **are representative of three experiment with similar results. (**E**) Expressions of IL-17, IL-32 and tartrate-resistant acid phosphatase (TRAP) in the synovium of the CIA and *IL-1Ra*-knock-out (KO) mice, two autoimmune arthritis mouse models. The results shown are representative of five experiments with similar results.

IL-17 and IL-32 interact in human FLSs and T cells from patients with RA. To confirm this interaction in autoimmune arthritis mouse models, splenic CD4^+ ^T cells of type-II collagen-induced mice (CIA) were cultured with anti-CD3 or anti-CD3 with IL-32α. Increased IL-17 secretion was observed with IL-32 stimulation in this animal model (Figure 4A). In addition, we examined whether IL-32-treated Th17 polarized cells secreted more IL-17, in a manner similar to that observed in the human RA condition. CD4^+ ^T cells and irradiated CD4^- ^T cells (as antigen presenting cells) from CIA mice at 5 weeks after immunization were co-cultured with Th17 polarizing condition with/without CII/IL-32. A second challenge with antigen increased CD4^+^IL-17^+ ^cells (Figure 4B). Moreover, IL-32 induced the secretion of IL-17 (Figure 4C) and increased the expression of IL-17 mRNA (Figure 4D). These results show that a second collagen challenge induced IL-17 secretion, and that IL-32 also promoted IL-17 mRNA expression and cytokine secretion.

Next, we investigated whether IL-17 and IL-32 have a role in bony erosion in RA mouse models. To correlate the location of cytokine expression and osteoclastogenesis, the synovium of both CIA and IL-1Ra knockout (KO) mice were analyzed for IL-17, IL-32 and TRAP expression, and stained with H&E (Figure 4E). Damaged bone areas demonstrated TRAP-positive cells. IL-32 and IL-17 were co-localized near the TRAP-positive areas between the cartilage and tarsal bone of both mouse models (Figure 4E). Thus, in two RA animal models, IL-32 induced IL-17 production, and IL-32 and IL-17 were expressed in areas of osteoclast differentiation.

### IL-17 and IL-32 synergistically induced osteoclastogenesis

**Figure 5:**
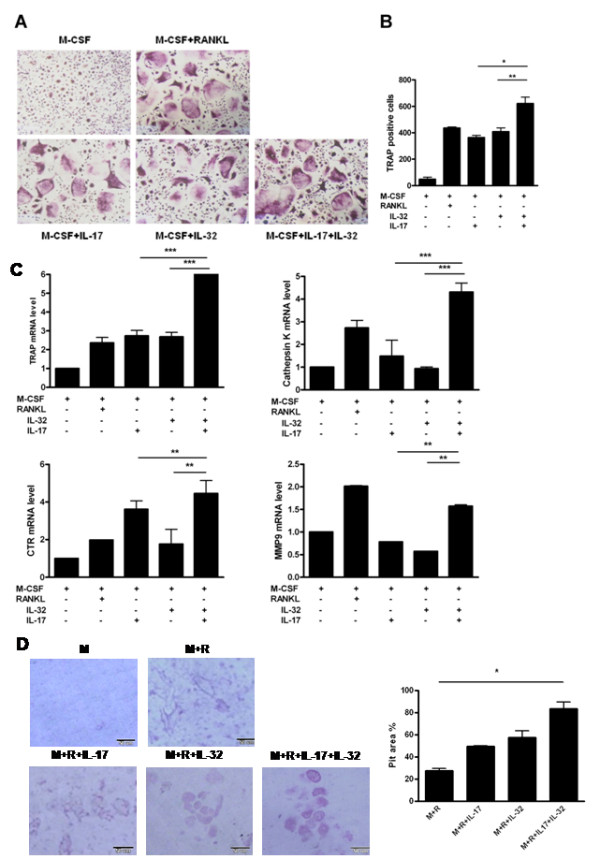
**Interleukin (IL)-17 and IL-32 synergistically induce osteoclastogenesis**. (**A**) Tartrate-resistant acid phosphatase (TRAP) staining for identification of osteoclasts. Osteoclast precursors were cultured in the presence of IL-17 (0.1 ng/ml) or/and IL-32 (5 ng/ml) with macrophage colony-stimulating factor (M-CSF; 25 ng/ml). The receptor activator of the nuclear factor kappa-B ligand (RANKL; 30 ng/ml)-treated group was the positive control. The medium and stimulus were changed every 3 days. After 15 days, the cells were stained for TRAP activity. (**B**) Numbers of multinucleated TRAP-positive cells per well. TRAP-positive cells containing two or more nuclei were scored as osteoclasts. TRAP-positive cells were counted three times by blind scoring. *P < 0.05 (compared with M-CSF+IL-32+IL-17), **P < 0.01 (compared with M-CSF+IL-32+IL-17). (**C**) The mRNAs of TRAP, Cathepsin K, calcitonin receptor (CTR) and matrix metallopeptidase 9 (MMP9), as osteoclast markers were quantified by real-time PCR. *P < 0.05 (compared with M-CSF+IL-32+IL-17), **P < 0.01 (compared with M-CSF+IL-32)+IL-17), ***P < 0.001 (compared with M-CSF+IL-32+IL-17). (**D**) Formation (left) and percent area (right) of resorption pits by osteoclasts on dentine discs. Cell culture was performed as described with dentine discs in 96-well plates. The osteoclast precursors were cultured in the presence of IL-17 (1 ng/ml) or IL-32 (5 ng/ml) with M-CSF (25 ng/ml) and RANKL (30 ng/ml). At day 21, the cells were removed from dentine. Resorption area was evaluated by light microscopy and measured using the image analysis software. (**A**), (**B**) and (**D**) are representative of two or three experiments with similar results. (**C**) Means ± SD of more than three separate experiments.

Osteoclast precursors were cultured in the presence of IL-17 and/or IL-32 with M-CSF, a cytokine involved in the survival and proliferation of osteoclasts. After 15 days culture, IL-17 or IL-32 induced the formation of multinucleated TRAP positive cells as shown in Figure 5A. The numbers of IL-17 or IL-32-stimulated TRAP-positive MNCs were increased compared to those observed with M-CSF alone. Although RANKL is well known as a crucial factor for osteoclast differentiation, these results revealed that IL-32 and IL-17 are involved in RANKL-independent mechanisms of osteoclastogenesis. In addition, the simultaneous stimulation by IL-17 and IL-32 induced an increased number of multinucleated TRAP-positive cells (Figure 5B). These findings imply that IL-17 and IL-32 synergistically induced osteoclastogenesis from osteoclast precursor cells in the absence of a RANKL-RANK interaction. Osteoclast markers, such as calcitonin receptor, cathepsin K, TRAP and MMP9 mRNAs, were increased by IL-17 or IL-32 as much as that observed with RANKL stimulation (positive control). IL-17 and IL-32 had a synergistic effect on induction of osteoclast markers at the mRNA level in a RANKL-independent manner (Figure 5C). In conditions that included IL-32 and IL-17 without RANKL, resorption pit formation was not observed. When treated with RANKL, IL-32 and IL-17 synergistically accelerated the osteoclast resorption activity compared with IL-32 or IL-17 alone (Figure 5D). These results indicate that IL-32 and IL-17 could synergistically induce osteoclastogenesis independent of RANKL, and could synergistically induce resorption by osteoclasts in a RANKL-dependent manner.

## Discussion

IL-32 is a novel cytokine that has been reported to be an important player in the innate and adaptive immune response. Innate immune stimulation by the ligands of toll-like receptors (TLRs) can induce the expression of IL-32 in FLSs [27]. Recently, the expression of IL-32 was demonstrated in *Mycobacterium tuberculosis *infections, inflammatory bowel disease and influenza A virus infection, in addition to that in autoimmune disease. However, more research is required to explain the function of IL-32 in inflammatory disorders.

The expression and regulation of cytokines involved in RA pathogenesis have been studied extensively over the past two decades, which is possibly due to the relative ease of analyzing the synovium of RA patients. These investigations have broadened and deepened our understanding of the roles of several cytokines in RA pathogenesis. Furthermore, some studies, most notably those focusing on TNFα, IL-1, and IL-6, have shown promising ways to block the cytokines involved in RA pathogenesis. It is important to characterize the individual cytokines in relation to the pathogenesis of RA and understanding the interaction and cross-talk between these cytokines is critical. Cytokines can amplify other inflammatory cytokines and then compensate for this by increasing the production of pro-inflammatory cytokines. For instance, it has been shown that IL-32 is a potent inducer of TNF-α [28]. In addition, TNF-α is a potent inducer of endogenous IL-32 expression, and IL-32 itself prolongs TNF-α production, thus inducing an important autoinflammatory loop [20]. IL-17 is critical for RA pathogenesis and can induce increased expression of TNFα [25]. To-date, a direct interaction between IL-17 and IL-32 has not been reported, although one study implied their interaction by determining that these cytokines have common signal intermediates, p300 and DAPK-1 [22]. Another study demonstrated that IL-32γ-stimulated DCs could induce a Th17 response when co-cultured with CD4^+ ^T cells [29], implying that IL-32 affects IL-17 production independent of TNFα.

In this study, we examined the synergistic expressions of IL-17 and IL-32 and their possible interactions. Using microarray analysis, we showed that IL-17 induced gene expression in the FLSs of patients with RA (Table 1), and that IL-32 mRNA levels increased in a dose-dependent manner (Figure 1A). Because IL-32α mRNA expression depends on the PI3-kinase/NF-κB systems in human colonic subepithelial myofibroblasts [30], we investigated whether IL-17 is also dependent on the NF-κB/PI3-kinase pathways in FLSs. of patients with RA. It was confirmed that this induction occurred through the NF-κB and PI3kinase pathways by using specific inhibitors to block these pathways (Figure 1B). IL-32 induction by TNF-α, is mediated via the Syk/Protein Kinase Cδ/JNK signaling pathways [31]. The transcription and secretion of pro-inflammatory and anti-inflammatory cytokines have the complex signal pathways inside cells.

IL-32 expression in the synovium from patients with RA than in those with OA was increased (Figure 1C). The expression of IL-32 in synovium of patients with RA was also co-localized with IL-17, NF-κB, and PI3K. This result suggests that FLSs from patients with RA could interact with T cells, especially IL-17-producing Th17 cells, and direct contact could be important for this interaction.

We observed that co-culture of FLSs with CD4^+ ^T cells resulted in production of IL-17, which caused IL-32 expression in FLSs. This IL-32 induction was partially inhibited by blocking with anti-IL-17 antibodies (Figure 2A). Coculture of FLSs with the IL-17-rich supernatant from Th17-polarized cells resulted in a markedly increased IL-32 production by FLSs from RA patients. To determine whether IL-17 had a direct effect, we used the supernatant of the IL-17 blocked Th17 polarized cells. These results suggested that IL-17 is directly related to IL-32 production with the interaction of CD4^+ ^T cells and FLSs. Since IL-17 was generally not produced by FLSs, IL-17 secreted from CD4^+ ^T cells might induce IL-32 expression in FLSs. IL-17-rich supernatant induced more IL-32 mRNA expression. Although these inductions were arrested by anti-IL-17 antibody, IL-17-rich supernatant with anti-IL-17 antibody was more reduced in IL-32 mRNA expression than CD4^+^T cell with anti-IL-17 antibody. The reason could be due to the production of other factors than IL-17 by cell-cell contact. The identification of these factors would be of interest for future study. It has been reported that TNFα exhibits potent induction of IL-32 secretion in FLSs of patients with RA. One study showed that IL-17 is related to IL-32 expression in FLSs of RA patients [19]. In contrast to our results, this study reported that both IL-17A and IL-17F induced IL-32 to only a small extent. In addition, IL-17 attenuated the IL-32 expression induced by TNFα. IL-17, an important proinflammatory cytokine, is unlikely to have a paradoxically negative feedback in the autoinflammatory loop between IL-32 and TNFα. Therefore, we suggest that our results, showing that IL-17 amplified IL-32 expression in FLSs of patients with RA, are logically acceptable.

Human IL-32 recombinant protein has been utilized in mice [18,29], and so we determined whether IL-32 could induce IL-17 production in an autoimmune arthritis mouse model. Using ELISA and FACS analysis, we showed that IL-32 induced high IL-17 expression in splenic CD4^+ ^T cells of CIA mice. IL-32 induced the differentiation of CD4^+ ^T cells of RA model mice to Th17 cells and promoted IL-17 production. In addition, immunohistochemistry staining indicated that IL-17, IL-32 and TRAP were co-localized in the joints of CIA and IL-1R antagonist-deficient mice. These results suggest that an interaction between IL-32 and IL-17 exists in both autoimmune arthritis diseases and animal models, contributing to accelerated inflammation and bone destruction.

Next, we revealed that IL-17 and IL-32 have synergistic roles in osteoclastogenesis. Osteoclasts are multinucleated cells that are responsible for bone resorption and are derived from hematopoietic precursor cells that circulate in the blood. It is currently thought that two critical factors (M-CSF and RANKL) supplied by osteoblasts are essential for the differentiation and maturation of osteoclast precursors. Moreover, some researchers have reported that a direct interaction between osteoclast progenitors and osteoblasts is required for IL-17-induced osteoclastogenesis [5]. IL-17 dose-dependently induced the expression of osteoclast differentiation factor (ODF) mRNA in osteoblasts. IL-32 also promotes osteoclast differentiation and the expression of several specific markers of osteoclasts such as NFATc1, OSCAR and cathepsin K [23]. To determine whether IL-17 and IL-32 have a synergistic effect in osteoclastogenesis, we cultured osteoclast precursors with IL-17 and/or IL-32. IL-17 and IL-32 accelerated osteoclastogenesis compared with IL-17, IL-32 or RANKL stimulation alone (Figure 5A, B). We also examined the effect of IL-17 and IL-32 on osteoclast differentiation in the presence of anti-RANKL or anti-OPG antibodies, and observed that osteoclastogenesis occurred in a manner that was independent of RANKL or OPG (data not shown). In addition, we investigated whether IL-17- and IL-32-stimulated osteoclastogenesis was affected by TNFα. Although we detected TNFα in the supernatant of IL-17- and IL-32-stimulated differentiated osteoclasts, blocking with anti-TNFα showed no effect (data not shown). Therefore, osteoclastogenesis associated with IL-17 and IL-32 was not dependent on RANKL or TNFα.

To determine the resorption activity of osteoclasts induced by IL-32 and Il-17, we performed a resorption pit assay using dentine slices. When IL-32 and IL-17 were assayed in the absence of RANKL, no resorption pit formation was observed. Moreover, we confirmed that RANKL is essential for osteoclast resorption activity. When treated with RANKL, IL-32 and IL-17 synergistically accelerated osteoclast resorption activity compared with IL-32 or IL-17 alone. We deduced that IL-32 and IL-17 could synergistically induce osteoclastogenesis independent of RANKL, and were synergistically involved in the RANKL-dependent resorption function of osteoclasts.

FLSs and CD4^+ ^T cells are found in close proximity in synovial joints. We suggested that IL-17 and IL-32 could stimulate the reciprocal production of each other, and amplify inflammatory reactions. In this model, the two cytokines synergistically stimulate osteoclastogenesis independently of RANKL, and might increasingly induce bony erosion and osteopenia together with RANKL, thus participating in the inflammation associated with RA. Therefore, interruption of IL-17 and IL-32 might be a therapeutic target for treatment of inflammatory arthritis.

## Conclusions

This report is the first to show that IL-17 induces IL-32 cytokine expression through the NF-κB and PI3-kinase signal pathways in FLSs of patients with RA. IL-17 is produced by Th17 cells that differentiate from CD4^+ ^T cells in patients with RA. In the joint environment of inflammation, CD4^+ ^T cells and FLSs interact with each other by direct contact and cytokine secretion, and this interaction amplifies the expression of IL-17 and IL-32. IL-32 induced high levels of IL-17 expression in the splenic CD4^+ ^T cells of CIA mice. Co-localization of IL-32, IL-17 and TRAP suggested the possibility of their functional interaction in autoimmune arthritis models. Both IL-17 and IL-32 induce the gene markers CTR, capthepsin K, TRAP and MMP9, which are all related with osteoclastogenesis. IL-17 and IL-32 have a synergistic effect on the expression of these genes in CD4^+ ^T cells and FLSs. IL-17 and IL-32 have a reciprocal influence on each other's production, and enhance osteoclastogenesis in the synovium of patients with RA.

## Abbreviations

ANOVA: analysis of variance; CIA: collagen-induced arthritis; CII: type II collagen; Cp: crossing point; DC: dendritic cell; FBS: fetal bovine serum; ELISA: enzyme-linked immunosorbent assay; FACS: fluorescence-activated cell sorter; FLS: fibroblast-like synoviocyte; H&E: hematoxylin and eosin; IFN: interferon; IL: interleukin; M-CSF: macrophage colony-stimulating factor; MMP: matrix metalloproteinase; MNC: multinucleated cell; NF-κB: nuclear factor-κB; NK: natural killer; OA: osteoarthritis; PI3K: phosphatidylinositol (PI)-3 kinase; PBMC: peripheral blood mononuclear cell; PCR: polymerase chain reaction; RA: rheumatoid arthritis; RANKL: receptor activator of nuclear factor kappa-B ligand; RBC: red blood cell; SDF: stromal cell-derived factor; TCR: T cell receptor; Th: T helper; TLR: toll-like receptor; TNF: tumor necrosis factor; TRAP, tartrate-resistant acid phosphatase.

## Competing interests

The authors declare that they have no competing interests in relation to this manuscript.

## Authors' contributions

YMM, BYY and MLC contributed to the conception and design, acquisition of data, analysis and interpretation of data, drafting of the article and final approval of the submitted manuscript. OHJ, KWK, KSP and SHP contributed to immunohistochemistry and TRAP staining. YJW, SYL, YMH, JSL and HYK helped with the PCR, FACS and ELISA. YMH contributed cell culture. All authors approved the final manuscript.
